# Prevalence and Reasons for the Absence of Vaginal Intercourse in Chinese Middle-Aged and Elderly Men

**DOI:** 10.1016/j.esxm.2022.100511

**Published:** 2022-04-12

**Authors:** Yi Lu, Jianzhong Zhang, Chengquan Ma, Hao Su, Hongjun Li

**Affiliations:** 1 Department of Urology, Peking Union Medical College Hospital, Peking Union Medical College, Chinese Academy of Medical Sciences, Beijing, China; 2 Department of Urology, Beijing Friendship Hospital, Capital Medical University, Beijing 100050, People's Republic of China

**Keywords:** Sexuality, Prevalence, China, Vaginal Intercourse, Aging

## Abstract

**Introduction:**

As the global population ages, research on the health of middle-aged and elderly men has intensified.

**Aim:**

To report a paucity of data on the prevalence, etiology, and risk factors associated with lack of vaginal intercourse in middle-aged and elderly Chinese men.

**Methods:**

Between January, 2018, and May, 2020, 6,096 men aged 40-90 years old who reside in mainland China were included in the community-based study. Validated scales related to erectile dysfunction (EHS and IIEF-5) and late-onset hypogonadism (ADAM and AMS), and in-person interview method were used to collect data. Multivariable analysis was performed to examine the risk factors associated with the absence of intercourse.

**Outcomes:**

Prevalence estimate of no current vaginal intercourse and its associations with basic factors and medical comorbidities. Reasons for no current vaginal intercourse.

**Results:**

The prevalence of intercourse absence was 19.8% (95% CI; 18.8–20.8%) in middle-aged and elderly Chinese males, and this proportion was significantly higher in older age groups (8.6%, 11.5%, 24.1%, and 34.2% for men aged 40–49, 50–59, 60–69, and 70–90, respectively; *P* < .05). Among the men who attributed the lack of intercourse to themselves, 168 (21.4%, 95% CI; 18.7–24.4%) had erectile problems and were apprehensive about erectile failure during intercourse. Strained spousal relationships (35 men, 8.4%, 95% CI; 6.1–11.4%), marital issues (163 men, 39%, 95% CI; 34.4–-43.8%), and poor health of the partner (179 men, 42.8%, 95% CI; 38.2–47.6%) were described as reasons for lack of intercourse with spouses. Same risk factors were also found in the multivariate analysis.

**Clinical implications:**

Modifiable factors that are related to lack of intercourse may be beneficial to Chinese middle-aged and elderly men.

**Strength & limitations:**

The main strength of the study is that it involved real-world settings. The limitations are as follows. Firstly, psychological data, data on sexual frequency and data regarding types of sex other than vaginal intercourse were not recorded. Secondly, this is a cross-sectional study, from which definite or causative conclusions can't be drawn. Thirdly, the spouses of the participants were not included in the study, and hence the data represent the perceptions of males only. Finally, objective data are required.

**Conclusion:**

Modifiable factors related to both the patients and their partners were associated with an increased rate of no intercourse in Chinese middle-aged and elderly men. Guidance for sexual life may benefit men with an absence of intercourse. Future studies are warranted to reexamine our findings.

**Lu Y, Zhang J, Ma C, et al. Prevalence and Reasons for the Absence of Vaginal Intercourse in Chinese Middle-Aged and Elderly Men. Sex Med 2022;10:100511**.

## INTRODUCTION

Although much is known about sexual dysfunction in men, there is little information about sexuality, especially among middle-aged and elderly men.^1^^,^^2^ A previously published study showed that despite older men showing a significant decline in sexual function, they were sexually active. Yet, their concerns regarding sexuality-related issues were rarely discussed, and there was a lack of guidance and solutions from physicians.^3^ Decline in sexuality can be classified as either a decline in sexual desire or a decline in sexual frequency. The latter differs from sexual dysfunction because it is a natural process of decline which is potentially caused by the following reasons: (i) the aging process, and (ii) aging accompanied by physical and psychological diseases.^4^ Furthermore, a decline in sexuality can have a negative effect on physical and psychological health.^5^

Lack of vaginal intercourse, a specific type of decline in sexual frequency, reportedly affects ∼18.1% of Chinese males.^6^ Marriage, personal health status, and lifestyle are potential risk factors. However, there is no detailed information regarding why men have no intercourse and the risk factors associated with lack of intercourse. What is the prevalence of no intercourse in Chinese middle-aged and elderly men and what are the reasons for the phenomenon: socioeconomic traits, aging or diseases? To answer this question, we conducted a nationwide study to identify the prevalence of the phenomenon and analyze related factors. The data may be used to understand the epidemiology of no vaginal intercourse in China, and develop strategies, policies, measurements, and health education programs to prevent the phenomenon of no intercourse, and provide potential targets for analyzing the etiology of no intercourse in future studies.

## MATERIALS AND METHODS

### Subjects

The participants for the community-based study were males of Chinese descent inhabiting mainland China. The study covered 29 provinces and autonomies in mainland China, excluding the Xinjiang autonomies, Qinghai Province, Tibet, Macao, and Hong Kong. We conducted a series of nationwide public lectures on men's health and asked whether the participants were willing to be enrolled in the study. We excluded men with psychiatric diseases and those who had severe diseases, such as organ failure and any malignancies. Subjects who consumed drugs that could affect sexual function, homosexuals, and virgins were excluded.

### Study Design and Procedures

Ethical approval adhering to the guidelines issued in the Declaration of Helsinki was obtained from the Medical Ethics Committee of the Peking Union Medical College Hospital (Study No. S-214, Beijing, China) as well as other participating institutes. Before enrollment, all subjects provided informed consent in writing, and we provided instructions to the participants to introduce the aim and details of the research.

The survey consisted of several validated questionnaires that included the following queries: (i) basic characteristics (eg, age, job, marital status, daily activity, etc.); (ii) common health status (eg, past and present medical history, drug intake history, etc.); (iii) inquiries about prostatitis (Have you ever had prostatitis? Is it cured? etc.); (iv) inquiries about intercourse (Do you still have vaginal intercourse? How long have you had no vaginal intercourse for? What are your reasons for lack of vaginal intercourse? etc.); (v) International Index of Erectile Function-5 (IIEF-5) for those who still have regular sexual intercourse^7^; (vi) Erection Hardness Score (EHS)^8^; (vii) androgen deficiency in the aging male (ADAM)^9^; and (viii) Aging Males’ Symptoms (AMS).^10^

Notably, individuals who reported no intercourse were interviewed in-person to obtain and reassure their reasons for no intercourse and for us to provide medical guidance for individuals. Some patients may misunderstand questions in the questionnaire, leading to categorization error and biases. Thus, the interview process included some questions and basic physical examinations was designed to verify information about personal health status and reasons reported in the questionnaire. The questions included erectile problems (yes or no), period of no intercourse, libido (high, average, low, or none), morning or nocturnal erection (yes or no), and reasons for no intercourse (attributable to himself or the partner). To provide hints for the subjects, we listed a few common reasons including erectile dysfunction, poor health, divorce, and poor spousal relationship.

### Diagnostic Criteria

We used the IIEF-5, a 5 item questionnaire, to evaluate erectile function, intercourse satisfaction, orgasmic function, sexual desire, and overall satisfaction of participants who had regular sexual intercourse in the last 6 months. A score of less than 22 was considered as ED. We used ADAM and AMS to assess the symptoms of late-onset hypogonadism (LOH) in participants. A positive ADAM was defined as a “yes” to decreased libido (question 1), erectile dysfunction (question 7), or the prevalence of any 3 or more other symptoms. An AMS score of > 26 was considered positive.

### Data Analysis

We analyzed data from the study using SPSS version 26.0 (SPSS Inc., Chicago, IL, USA). We calculated the prevalence estimates of no sexual intercourse and its variations in different age ranges. The *χ²* test was used to analyze raw and age-adjusted prevalence using demographic and medical information. Multivariate analysis was used to calculate age- and multivariate-adjusted odds ratios (ORs) and the 95% confidence intervals (CIs). A test for trend by increasing number of illnesses using logistic regression was performed. A 2 tailed *P* < .05, indicated statistical significance in all data analyses.

## RESULTS

Between January, 2018, and May, 2020, 6,393 surveys were collected and some of them were incomplete, were repetitions, or were otherwise invalid. Once these had been removed, the response rate was 95.4% (6096/6393). All 6096 participants were Chinese men aged between 40 and 90 years old, and none of them were virgins. A total of 1204 (19.8%, 95% CI; 18.86–20.8%) males reported a current lack of sexual intercourse lasting more than 1 year. The prevalence estimates of no intercourse was significantly higher in men with older age (*P* < .05) (Table 1). Particularly high estimates of no intercourse rate differences were observed in men ages 60-69 and 50–59 years (12.6%, 95% CI; 9.8–15.3%) and men ages 60-69 and 70-90 years (10.1%, 95% CI; 6.8–13.2%).

**Table 1:** Prevalence estimate of currently no virginal intercourse in 6,096 Chinese men

Age, years	No. of subjects	Percentage of subjects, %	No. of no sexual intercourse	Prevalence, %*
40-49	1519	25	130	8.6
50-59	1506	25	174	12
60-69	1481	24	357	24
70-90	1590	26	543	34

⁎ The prevalence was not adjusted by age.

Table 2 shows the raw and age-adjusted prevalence estimates for the current lack of intercourse based on the individuals’ basic information, medical history, and health condition. These results can be observed when comparing the raw and age-adjusted prevalence rates. Comparison of the normal body mass index (BMI) and waist-to-hip ratio (WHR) showed that the group with abnormal BMI and WHR showed a significantly higher rate of no intercourse (*P* < .05). Marriage (separated/divorced/widowed), spousal relationship (ordinary or poor), low education status, smoking, alcohol intake, lack of physical activity, the number of past and present illnesses, and a few andrological conditions (LOH, prostatitis, erectile problem, and low sexual desire) were associated with currently no intercourse (*P* < .05). Notably, the rate of no intercourse in recently remarried men was similar to that of married men (*P* > .05).

**Table 2:** Prevalence estimates of currently no intercourse by basic factors and medical comorbidities in 6,096 Chinese men

				Prevalence. %	
Items	Subjects	Age. Years*	No. of no sexual intercourse	Raw	Age adjusted
BMI					
<18	117	62 ± 11	24	21†	21†
[18, 25)	3301	49 ± 10	512	16	11
[25, 29]	2199	52 ± 10	409	19†	19†
>29	479	59 ± 10	259	54†	50†
WHR					
<0.9	3256	51 ± 11	515	16	13
≥ 0.9	2840	52 ± 11	689	24†	16†
Ethnicity					
Han	5720	51 ± 9	1121	20	15
Others	376	50 ± 8	83	22	16
Job					
Mental work	2794	50 ± 8	536	19	19
Physical work	2887	51 ± 9	578	20	20
Unknown	415	53 ± 10	90	22	21
Residence					
Urban	4665	51 ± 9	908	20	19
Rural	1431	50 ± 10	296	21	21
Marriage					
Married/cohabiting	5569	50 ± 8	826	15	15
separated/divorced/widowed	400	51 ± 9	359	90†	85†
Digamous	127	53 ± 11	19	15	16
Spouse relationship					
Good	3730	51 ± 9	520	14	11
Ordinary	2070	51 ± 9	431	21†	24†
Bad	296	52 ± 11	253	86†	85†
Educational status					
Primary education	785	56 ± 10	348	44	50
Middle school	2732	50 ± 8	494	18†	14†
Higher education	2579	48 ± 9	362	14†	12†
Monthly income. CNY					
0-2000	1429	55 ± 9	418	29	29
2000-3999	2362	54 ± 9	428	18†	15†
4000-5999	1518	50 ± 11	265	18†	16†
6000-7999	425	49 ± 9	54	13†	11†
8000-	362	48 ± 9	39	11†	12†
Smoking					
Yes	1983	51 ± 9	560	28	25
No	4113	51 ± 10	644	16†	13†
Alcohol intake					
Yes	1953	51 ± 9	329	17	17
No	5399	50 ± 9	805	15†	15†
Exercise					
Yes	2523	51 ± 9	429	17	14
No	3573	50 ± 9	775	22†	20†
No. of past illness					
0	3280	48 ± 10	423	13	12
1	2242	50 ± 9	539	24†	26†
2	454	54 ± 10	116	26†	26†
3	112	58 ± 12	79	71†	67†
4	8	64 ± 12	7	88†	88†
No. of present illness					
0	2931	46 ± 9	320	11	8.8
1	2372	50 ± 10	570	24†	24†
2	548	54 ± 9	178	33†	35†
3	188	59 ± 10	91	49†	45†
4	43	60 ± 8	34	79†	82†
5	13	64 ± 10	10	77†	73†
6	1	69	1	100†	100†
Prostatitis history					
Yes	1590	53 ± 11	436	27	30
No	4506	50 ± 11	768	17†	14†
ADAM					
Positive	4437	56 ± 11	964	22	22
Negative	1659	49 ± 10	240	15†	15†
AMS					
Positive	4429	57 ± 11	1000	23	20
Negative	1667	50 ± 10	204	12†	13†
Sexual desire					
High	746	44 ± 7	73	9.8	9.9
Normal	3255	50 ± 11	466	14†	15†
Low/None	2095	55 ± 12	665	32†	29†
Erection problem					
Yes	3076	58 ± 11	956	31	34
No	3020	43 ± 11	248	8.2†	7.6†
Nocturnal or morning erection					
Yes	3428	57 ± 10	386	11	9.0
No	2668	43 ± 10	808	30†	34†

⁎ Age is presented as mean ± SD.

† *P* < .05 in comparison within subgroup. ADAM = Androgen Deficiency in the Aging Male; AMS = The Aging Males' Symptoms; BMI, body mass index; CI = confidence interval; CNY = China Yuan; OR = odds ratio; Ref = reference; WHR = waist-hip ratio.

**Figure 1:**
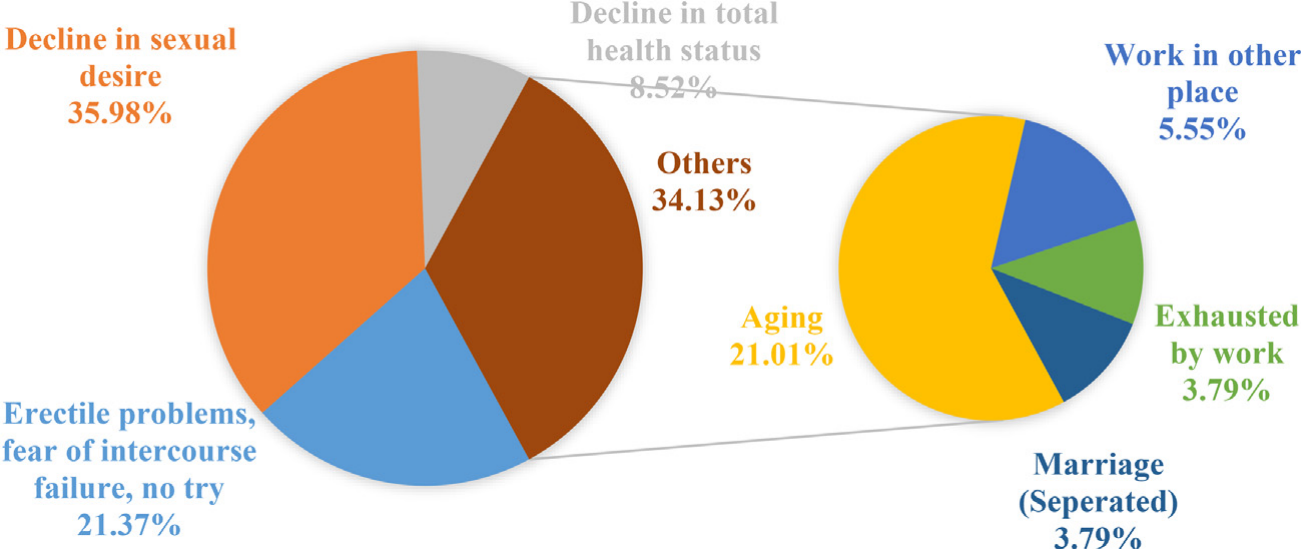
Reasons reported for lack of current vaginal intercourse attributed to the self by the participant.

**Figure 2:**
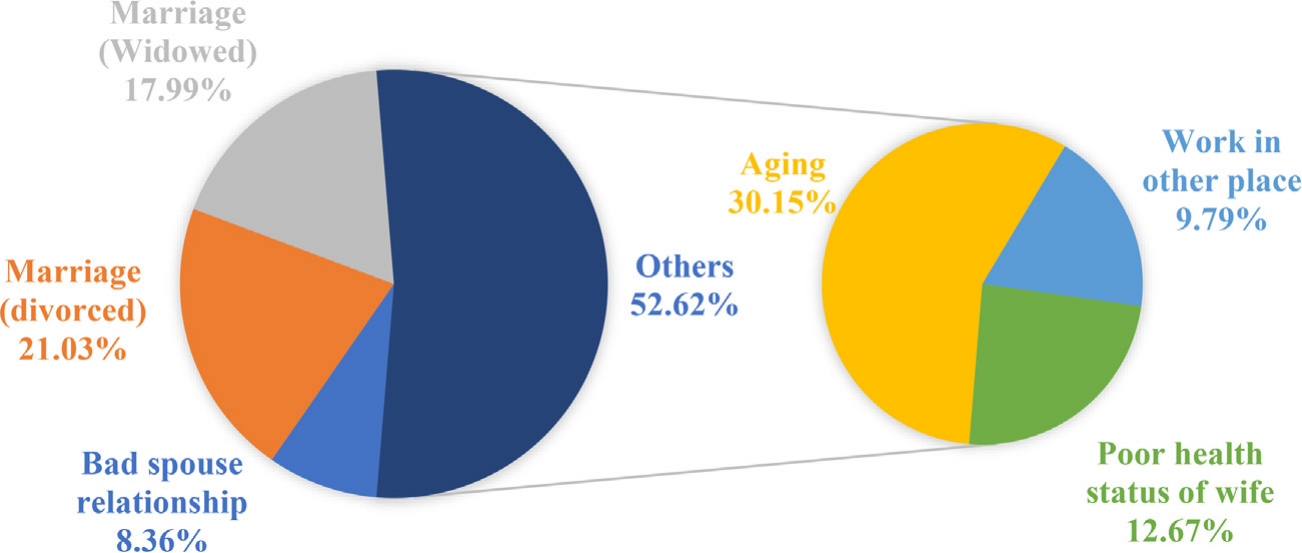
Reasons reported for lack of current vaginal intercourse attributed to the spouse by the participant.

Etiological analysis results from the in-person interview of the study indicated that 786 (65.3%) men attributed the reasons for currently no intercourse to themselves, whereas 418 (34.7%) men attributed it to their sexual partners (Table A.1). Among the reasons identified as self-caused, a decline in sexual desire (283, 36%, 95% CI; 32.7–39.4%); erectile problems, fear of intercourse, lack of attempts (168, 21.4%, 95% CI; 18.7–24.4%); decline in total health status (67, 8.5%, 95% CI; 6.8–10.7%); and other reasons (269, 34.2%, 95% CI; 31–37.6%), including aging (165, 21%, 95% CI; 18.3–24%), working in another place (44, 5.6%, 95% CI; 4.2–7.4%), exhaustion due to work (30, 3.8%, 95% CI; 2.7–5.4%), and marital separation (30, 3.8%, 95% CI; 2.7–5.4%) were identified. Among spouse-related reasons, poor spousal relationship (35, 8.4%, 95% CI; 6.1–11.4%), divorce (88, 21.1%, 95% CI; 17.4–25.2%), widowhood (75, 18%, 95% CI; 14.6–21.9%), and other reasons (220, 52.6%, 95% CI; 47.8–57.4%), including aging (126, 30.1%, 95% CI; 26–34.7%), work in other places (41, 9.8%, 95% CI; 7.3–13%), and poor health status of the spouse (53, 12.7%, 95% CI; 9.8–16.2%) were identified as contributing factors (Figures 1 and 2).

Age and multivariate-adjusted ORs are shown in Table A.2, which indicates whether listed factors were risk factors for no intercourse. The age-adjusted analysis results indicated that abnormal BMI (low or high), abnormal WHR, unharmonious marriage and spousal relationship, low education level and income, alcohol and cigarette intake, lack of physical exercise, the prevalence of diseases in the past or present, andrological conditions (LOH, prostatitis, erectile problem, and low sexual desire) (*P* < .05), and remarriage were not associated with an increased risk of no intercourse (*P* > .05). The same risk factors were also identified in the multivariate adjustment model.

## DISCUSSION

In this study, we reported the prevalence of no current vaginal intercourse, analyzed the reasons, and investigated the risk factors associated with no intercourse in middle-aged and elderly Chinese men. Our findings, based on representative population data from China,^6^^,^^11^^,^^12^ indicate that the overall rate of no vaginal intercourse in Chinese men aged 40–90 years is 19.8% (95% CI; 18.8–20.8%). Accordingly, results from in-person interviews indicated that male erectile problems, a decline in sexual desire, fear of failure during intercourse, and poor spousal relationship were reasons for no intercourse, whereas remarriage was not. Consistent results were identified in the multivariate analysis, which showed that the BMI, WHR, marriage and spousal relationship, education status, cigarette and alcohol intake, physical activity, number of past and present illnesses, and certain andrological conditions (LOH, prostatitis, erectile problem, and low sexual desire) are associated with no intercourse and can be considered as risk factors.

Current studies on sexuality have focused on people with sexual dysfunction, diverse sexual behaviors, or those who have high-risk sex,^3^^,^^13^ and there are few reports on no intercourse and analysis of its causes, especially in middle-aged men and elderly men. The results indicating the prevalence of no sexual intercourse in Chinese men at a rate of ∼19.8% are similar to those of a previous study (18.1%), mentioned as a secondary outcome of the study conducted by Zhang et al. in 2017.^6^ Zhang and colleagues selected 5,210 subjects who were over 40 years old, and differences in sample selection and exclusive criteria may have led to the difference in rates observed. We identified higher no intercourse rates in participants with older ages; however, 65.8% of subjects in their 70s and 75.9% in their 60s were engaged in vaginal intercourse. Figure A.1 indicated that the percentage of lack of sexual function is robust among age subgroups, while the percentage of men attributing absence of intercourse to aging was significantly higher in older men. It can be inferred that sexuality is still essential and important to older men, and opinions on this view are similar.^3^^,^^14^^,^^15^

The risk factors for no intercourse in men included behaviors in daily routine, body measurement indices, socioeconomic factors, and medical history, including men's health-related diseases. The multi-cause effect correlates with the multi-dimensional feature of sexuality^16^^,^^17^ and may indicate the role of sexual intercourse in overall health and emphasize the non-biological significance of sexuality. Physical factors play a central role in sexual dysfunction and lack of sexual intercourse. Smoking, drinking, lack of exercise, central obesity, and diet are well-established risk factors for sexual dysfunction and a decline in sexuality in men that regularly have sex.^18^, ^19^, ^20^, ^21^ Marital status, degree of intimacy, income, and education have also been described in previous studies in association with their effects on sexuality.^16^^,^^22^ The change from normal sexuality to decline in sexuality or sexual dysfunction to no intercourse can be observed as a gradual and continuous process. In line with these findings, we assessed the impact of lifestyle and socioeconomic factors on no intercourse in men and found that these factors were also closely related to no intercourse. Socioeconomic development and spousal relationship improvements are likely to reduce the proportion of men with no intercourse.

By conducting multivariate analyses, we found that present and past health status, including some men's health indices, may increase the risk of no intercourse. Arne et al. reported that German men who described their health status as good showed significantly reduced rates of sexual activity compared with men with fair or bad health.^23^ Comparable results were observed in men with chronic diseases and disabilities, which may negatively affect sexuality.^23^ In this study, we found that compared with men with no present or past diseases, men with 1 or more diseases showed a higher rate of no intercourse, and we observed a significant and gradual increase in no intercourse as the number of present and past diseases increased. Previous studies have reported associations between androgen or LOH, prostate diseases, erection problems, sexual desire, and sexuality.^24^, ^25^, ^26^, ^27^, ^28^, ^29^ The wide distribution of androgen receptors throughout the body indicates a crucial role for testosterone in sexuality, including both the excitation and inhibition of sex. It has been shown in several studies that LOH is characterized by diffuse symptoms of androgen deficiency, including sexual difficulties or dysfunction, muscle weakness, obesity, and fatigue, among others. Costa et al. showed the potentially direct role of sexual symptoms for LOH in sexual satisfaction.^28^^,^^30^^,^^31^ Our findings may indicate an association between the effect of sexual symptoms of LOH on sexual behavior. Prostatitis, often accompanied by lower urinary tract symptoms (LUTS), is a confusing disease that occurs in men at ages before benign prostatic hyperplasia (BPH) becomes universal. In 2009, Liang et al. reported that 1,071 (8.4%) of 12,743 Chinese men had prostatitis symptoms, and 571 (4.5%) were finally diagnosed with chronic prostatitis.^32^ In our study, men with prostatitis showed a higher rate of no intercourse, which may be caused by the aggravated pain induced by contraction of the prostate when ejaculation occurs. Men with prostatitis may reduce the frequency of sex or even prefer sexual abstinence to mitigate pain. Furthermore, some doctors may ignore the role of behavior improvement, such as a guide for intercourse in the treatment of prostatitis.^33^ However, it should be noted that the rationale for men to avoid sexual intercourse when diagnosed with prostatitis is still unclear, especially for chronic prostatitis.^34^ Clinical practitioners are responsible for guiding patients’ behavior and habits to facilitate treatment outcomes.

Our findings have practical implications. First, we analyzed the reasons for no intercourse and identified significant risk factors associated with no vaginal intercourse in a clinical setting, although the underlying mechanisms should be explored further. Second, some of the risk factors are highly modifiable, such as alcohol and cigarette intake, lack of exercise, LOH, remarriage, treatment for diseases of the spouse, lack of sexual guidance and encouragement, and sexual dysfunction, which should be considered in future prevention measures. Third, socioeconomic status can affect human mortality, longevity, and self-rated health through lifestyle factors.^35^^,^^36^ Our study shows that socioeconomic status can also influence sexuality and sexual behavior. Therefore, solutions to reduce socioeconomic inequalities may be beneficial to men's health.

The main strength of the study includes the real-world settings; we did not set many restrictions on the subjects and conducted multivariate analysis to draw our conclusions. Although our study is the first to focus on middle-aged and elderly men with no vaginal intercourse in China, certain limitations should be noted. Firstly, we did not collect psychological data, data on sexual frequency, or data on other types of sex, such as oral sex or anal intercourse. This information may help understand the questions better. Secondly, the reasons collected represent the perceptions of males only since their spouses were not participants, which brings inevitable bias. Thirdly, the nature of this cross-sectional study makes it difficult for us to arrive at definite or causative conclusions, although we provide some directions for future studies on lack of vaginal intercourse. Last but not the least, the study included many subjective indicators filled in by patients, although some objective scales (IIEF-5, AMS, etc.) were also used. In-person interviews were designed to further collect data on, and reassure reasons for, the absence of intercourse in individuals who reported no current intercourse.

## CONCLUSIONS

By performing a nationwide study, we found that the prevalence of no vaginal intercourse in middle-aged and elderly Chinese men was 19.8%. Most risk factors for the absence of vaginal intercourse are modifiable. Physicians should treat sexual dysfunction with efficient drugs, and also help patients and their partners establish a progressive view of male diseases and provide guidance for their sexual life.

## STATEMENT OF AUTHORSHIP

YL, JZZ, HS designed the study and JZZ and CQM collected data. YL, JZZ, and CQM carried out data analysis, and YL wrote the article and all authors revised the manuscript.

### Consent for Publication

Not applicable

### Availability of Data and Materials

The datasets generated during and/or analyzed during the current study are available from the corresponding author on reasonable request.

### Ethics Approval and Consent to Participate

The study (No. S-214) was approved by the medical ethic committee of the Peking Union Medical College Hospital. The study was conducted in compliance with the principles of the Declaration of Helsinki, the principles of Good Clinical Practice and in accordance with all applicable regulatory requirements.

This manuscript involves human participants. The informed consent was obtained from each participant.

## Appendix Supplementary materials

Image, application 1
