# Supplementary material for: Prevalence and Reasons for the Absence of Vaginal Intercourse in Chinese Middle-Aged and Elderly Men
Source: Sex Med. 2022 Apr 12;10(3):100511. doi: 10.1016/j.esxm.2022.100511 (PMC9177884; doi:10.1016/j.esxm.2022.100511)

Table A.1. Reasons for currently no intercourse.

	Reasons	N
**Himself**		**786**
	Erectile problems, fear of intercourse failure, no try	168
	Decline in sexual desire	283
	Decline in total health status	67
Other reasons		269
	Aging	165
	Work in other places	44
	Exhausted by work	30
	Marriage (Seperated)	30
**Wife**		**418**
	Bad spouse relationship	35
	Marriage (divorced)	88
	Marriage (Widowed)	75
Other reasons		220
	Aging	126
	Work in other places	41
	Poor health status of wife	53

Table A.2. Age- and multivariable-adjusted ORs (95% CIs) for currently no intercourse by basic features and chronic diseases in Chinese men at least 40 years old

	Age-adjusted		Multivariate-adjusted	
Items	OR	95%CI	OR	95%CI
Age	—	—	3.23	2.87-3.69
BMI				
<18	2.12	1.35-3.34	2.01	1.30-2.79
[18, 25)	1.00 (Ref)		1.00 (Ref)	
[25, 29]	1.78	1.55-2.04	1.66	1.45-1.97
>29	1.43	1.09-1.86	1.48	1.13-1.91
WHR				
< 0.9	1.00 (Ref)		1.00 (Ref)	
≥ 0.9	1.43	1.25-1.62	1.30	0.92-1.81
Ethnicity				
Han	1.00 (Ref)		1.00 (Ref)	
Others	1.07	0.83-1.39	1.02	0.72-1.38
Job				
Mental work	1.00 (Ref)		1.00 (Ref)	
Physical work	1.07	0.94-1.22	1.03	0.92-1.13
Unknown	1.12	0.87-1.44	1.10	0.85-1.35
Residence				
Urban	1.00 (Ref)		1.00 (Ref)	
Rural	1.11	0.96-1.29	1.08	0.79-1.38
Marriage				
Married/cohabiting	1.00 (Ref)		1.00 (Ref)	
Separated/divorced/Widowed	6.37	5.12-7.93	5.94	5.02-7.06
Digamous	1.62	0.97-2.46	1.39	0.91-1.87

Spouse relationship				
Good	1.00 (Ref)		1.00 (Ref)	
Ordinary	1.33	1.15-1.54	1.42	1.15-1.70
Bad	19.74	15.41-25.29	19.39	15.49-23.25
Educational status				
Primary education	1.00 (Ref)		1.00 (Ref)	
Middle school	0.39	0.33-0.46	0.42	0.34-0.50
Higher education	0.33	0.27-0.39	0.36	0.29-0.43
Monthly income. CNY				
0-2000	1.00 (Ref)		1.00 (Ref)	
2000-3999	0.75	0.64-0.88	0.83	0.52-1.13
4000-5999	0.70	0.59-0.84	0.77	0.53-1.07
6000-7999	0.53	0.40-0.72	0.65	0.48-0.83
8000-	0.42	0.30-0.60	0.57	0.41-0.73
Smoking				
Yes	1.00 (Ref)		1.00 (Ref)	
No	0.59	0.52-0.67	0.65	0.51-0.78
Alcohol intake				
Yes	1.00 (Ref)		1.00 (Ref)	
No	0.89	0.75-1.00	0.80	0.73-0.88
Exercise				
Yes	1.00 (Ref)		1.00 (Ref)	
No	1.19	1.04-1.35	1.20	1.08-1.33
No. of past illness				
0	1.00 (Ref)		1.00 (Ref)	

1	2.94	2.54-3.40	2.16	1.88-2.43
2	3.27	2.63-4.07	3.03	2.71-3.34
3	4.03	2.67-6.07	4.01	2.84-5.20
4	22.02	5.67-85.51	18.33	7.09-31.21
P_{trend}	< 0.001		< 0.001	
No. of present illness				
0	1.00 (Ref)		1.00 (Ref)	
1	2.56	2.21-2.97	2.60	2.19-3.02
2	3.38	2.73-4.17	3.19	2.79-3.64
3	5.73	4.27-7.70	5.68	4.31-7.09
4	11.16	6.40-19.47	10.65	6.87-14.51
5	12.06	4.03-36.09	12.58	4.76-20.37
6	—	—	—	—
P_{trend}	< 0.001		< 0.001	
Prostatitis history				
Yes	1.00 (Ref)		1.00 (Ref)	
No	0.66	0.57-0.75	0.72	0.60-0.85
ADAM				
Positive	1.00 (Ref)		1.00 (Ref)	
Negative	0.50	0.42-0.58	0.58	0.48-0.69
AMS				
Positive	1.00 (Ref)		1.00 (Ref)	
Negative	0.44	0.38-0.53	0.55	0.40-0.70
Sexual desire				
High	1.00 (Ref)		1.00 (Ref)	

Normal	1.60	1.23-2.07	1.63	1.24-2.00
Low/None	3.76	2.90-4.87	3.66	2.76-4.56
Erection problem				
Yes	1.00 (Ref)		1.00 (Ref)	
No	0.22	0.19-0.26	0.42	0.22-0.63
Nocturnal or morning erection				
Yes	1.00 (Ref)		1.00 (Ref)	
No	3.11	2.72-3.56	3.02	2.58-3.47

Abbreviations: Ref: reference, OR: odds ratio, CI: confidence interval, BMI: body mass index, WHR: waist-hip ratio, CNY: China Yuan, ADAM: Androgen Deficiency in the Aging Male, AMS: The Aging Males' Symptoms.

Fig. A.1. Age subgroup analysis for reasons of absence for no intercourse.

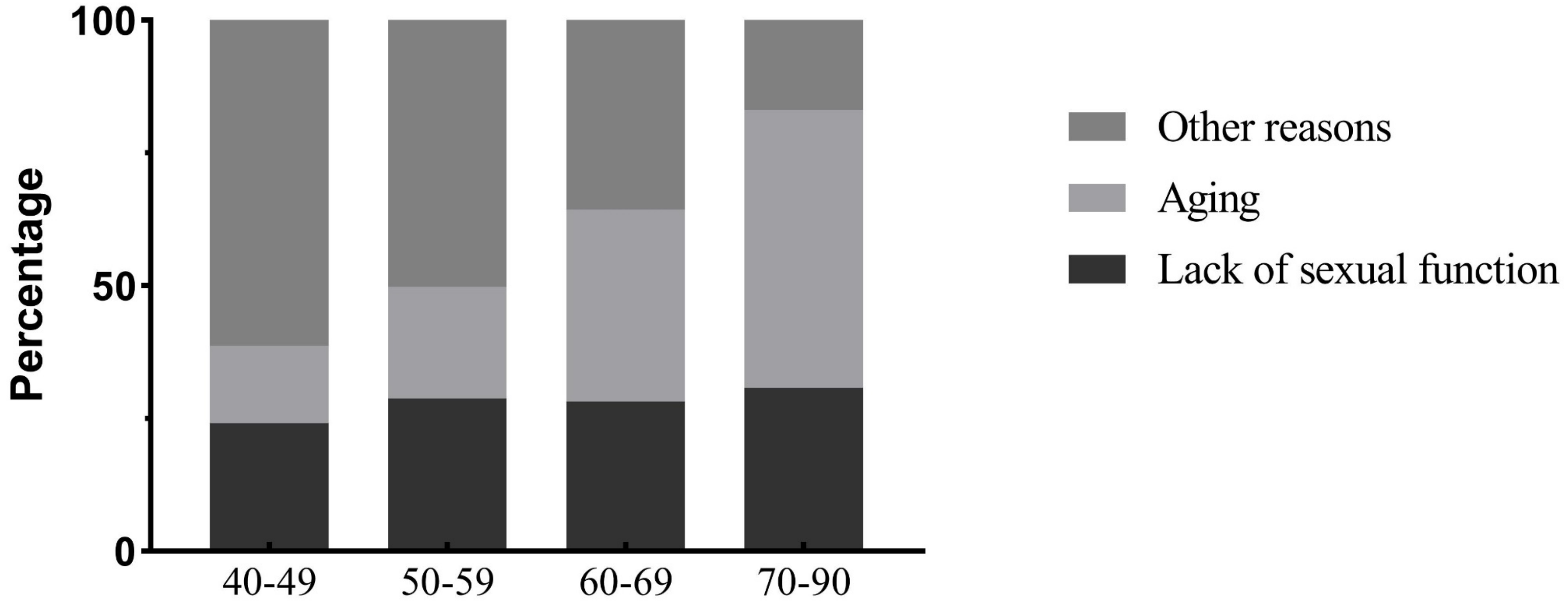

Supplement: Supplementary file 1 [file mmc1.pdf]
